# Supplementary figures and images for: Contributions of human amygdala nuclei to resting-state networks
Source: PLoS One. 2022 Dec 28;17(12):e0278962. doi: 10.1371/journal.pone.0278962 (PMC9797096; doi:10.1371/journal.pone.0278962)

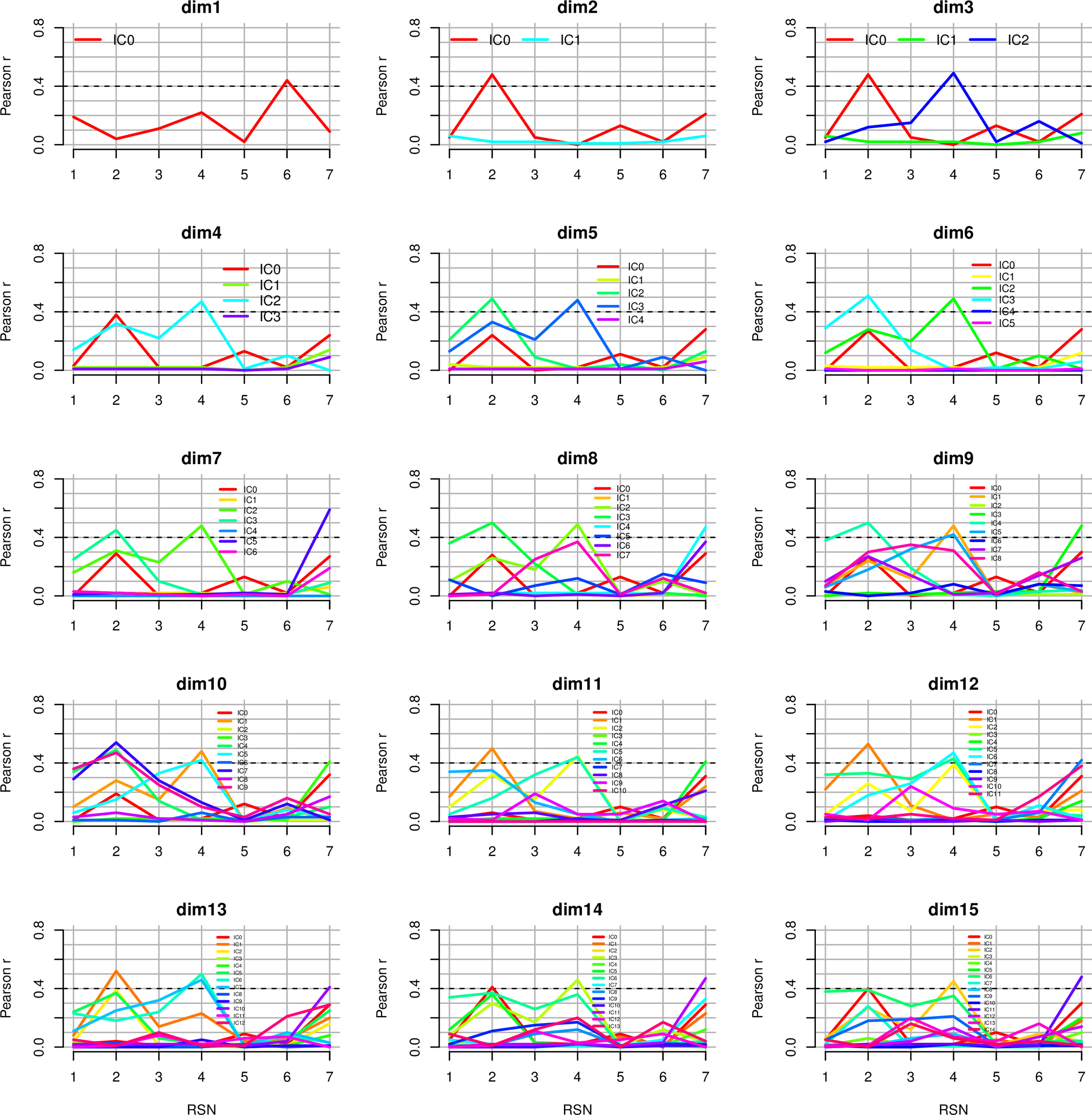

Supplement: S1 Fig — Note that three Yeo networks (2,4,7) appear frequently across all dimensions and that the lowest dimension at which these three networks are detected with good strength and separability is dimension 7. (TIF) [file pone.0278962.s001.tif]

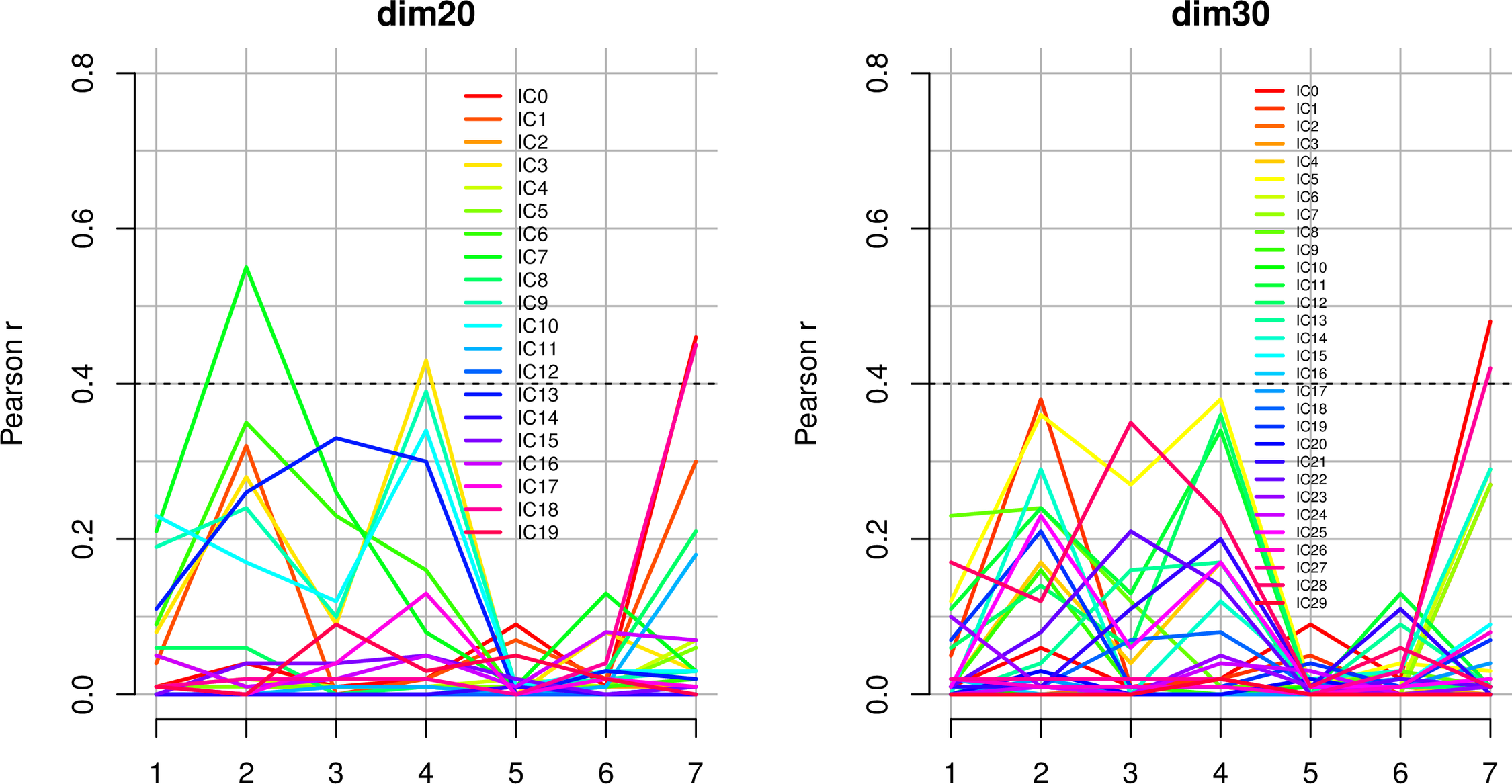

Supplement: S2 Fig — Note how even at increased dimensions, networks 2 (somatomotor), 4 (ventral attention) and 7 (default mode) seem most frequently detected, albeit less reliably. (TIF) [file pone.0278962.s002.tif]

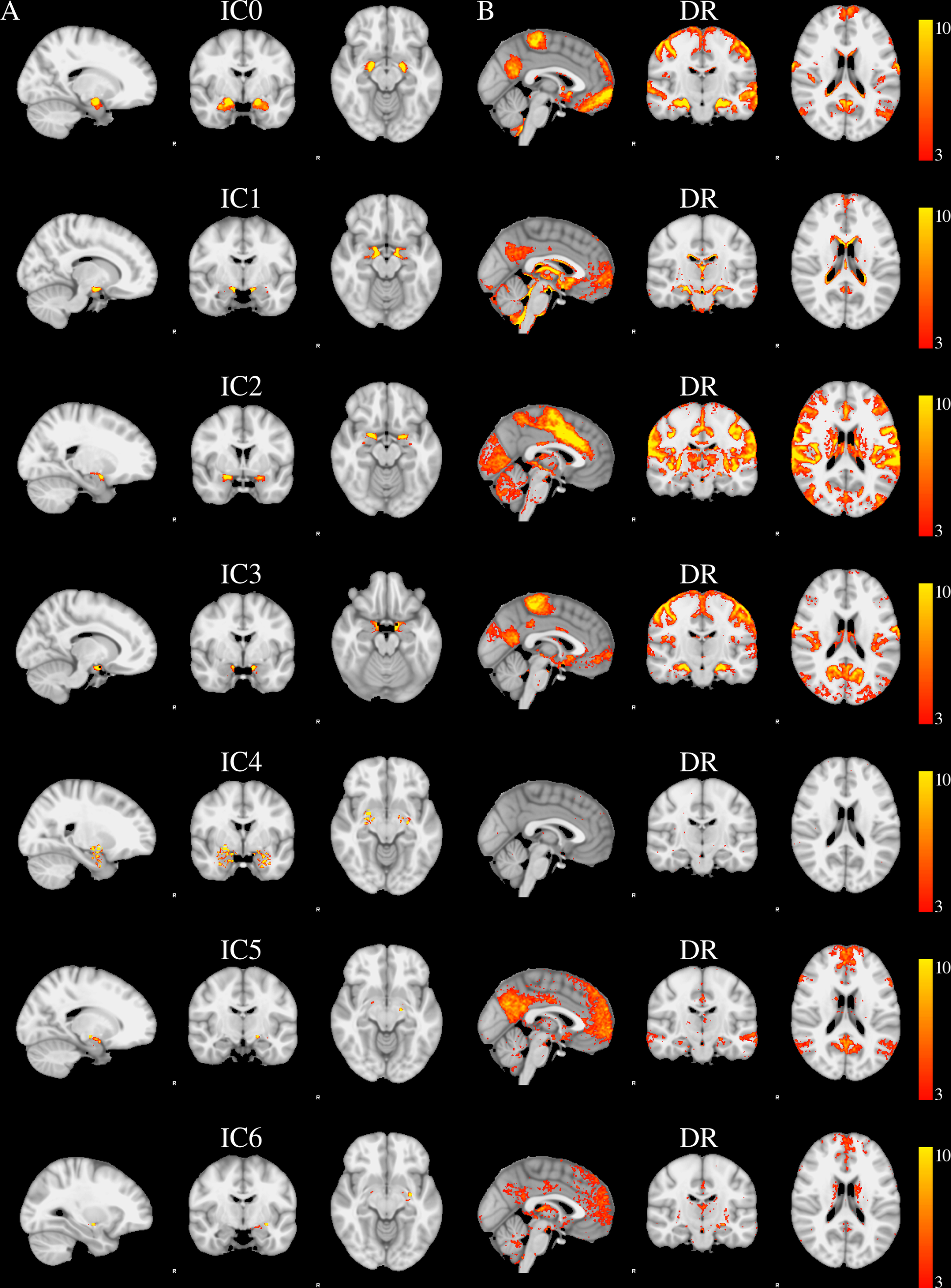

Supplement: S3 Fig — Note ICs 2 (Ventral Attention), 3 (Somatomotor), and 5 (default mode) were detected by the algorithm as having a strong and unique relationship with the Yeo networks (see main manuscript text for details). (TIF) [file pone.0278962.s003.tif]

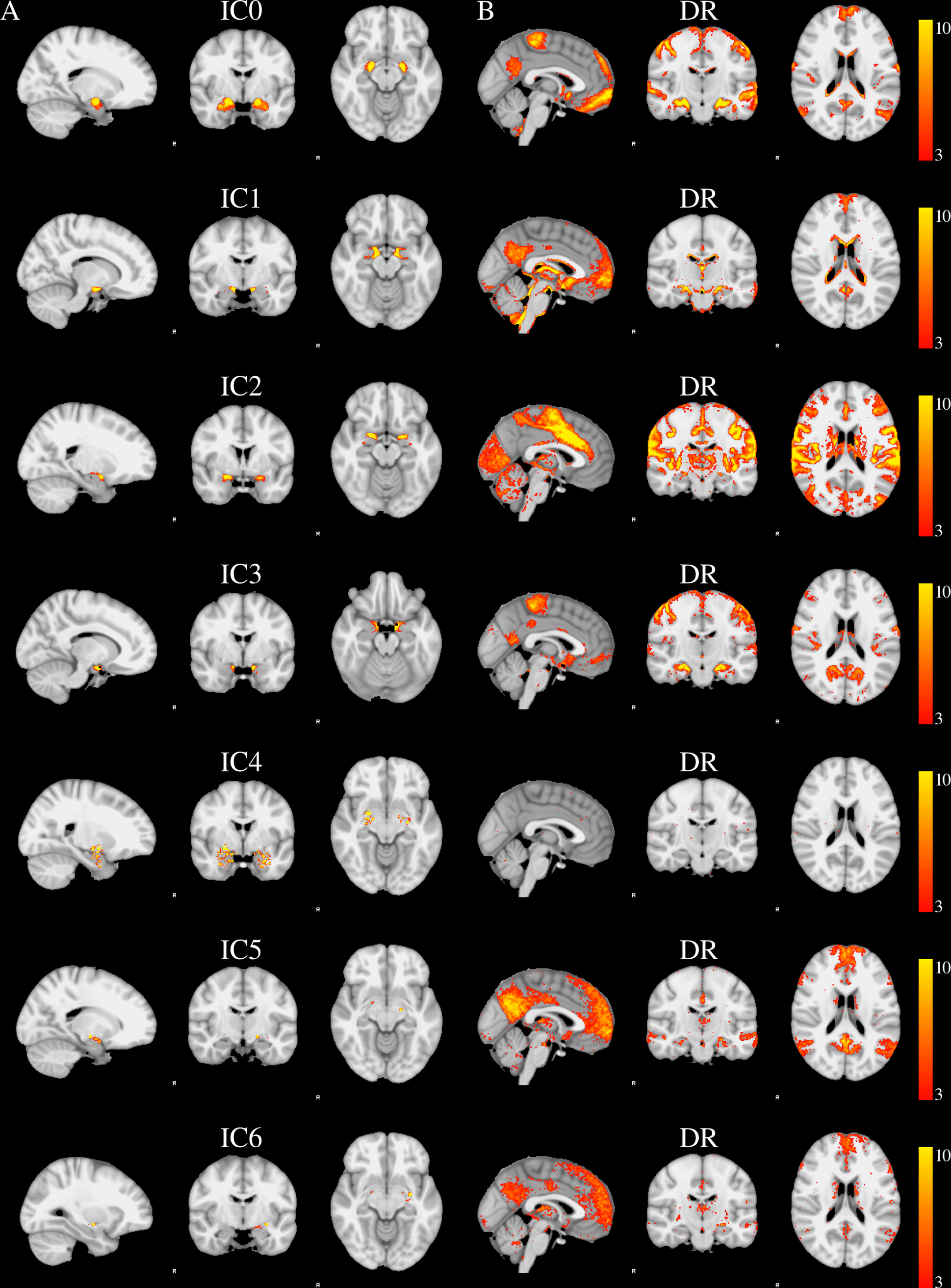

Supplement: S4 Fig — Note the high similarity between the obtained functional connectivity for ICs 2, 3 and 5 in the validation and test datasets (cf., S3 Fig). (TIF) [file pone.0278962.s004.tif]
