# Supplementary material for: Contributions of human amygdala nuclei to resting-state networks
Source: PLoS One. 2022 Dec 28;17(12):e0278962. doi: 10.1371/journal.pone.0278962 (PMC9797096; doi:10.1371/journal.pone.0278962)
Supplement: S1 File — (PDF) [file pone.0278962.s005.pdf]

Supplementary Materials for Contributions of human amygdala nuclei to resting-state networks

Uriel K. Arguinzones[1], Sara Seoane[1], Joost Janssen[2,3,4] and Niels Janssen[1,5,6,7]

[1]Department of Psychology, Universidad de la Laguna, Spain
[2]Department of Child and Adolescent Psychiatry, Institute of Psychiatry and Mental Health, Hospital General Universitario Gregorio Marañón, Spain
[3]Ciber del Área de Salud Mental, Instituto de Investigación Sanitaria Gregorio Marañón, Spain
[4]Department of Psychiatry, UMCU Brain Center, University Medical Center Utrecht, the Netherlands
[5]Institute of biomedical technologies, Universidad de La Laguna, Spain
[6]Institute of neurosciences, Universidad de la Laguna, Spain
[7]Department of Neurobiology and Behavior, University of California, Irvine, California, USA

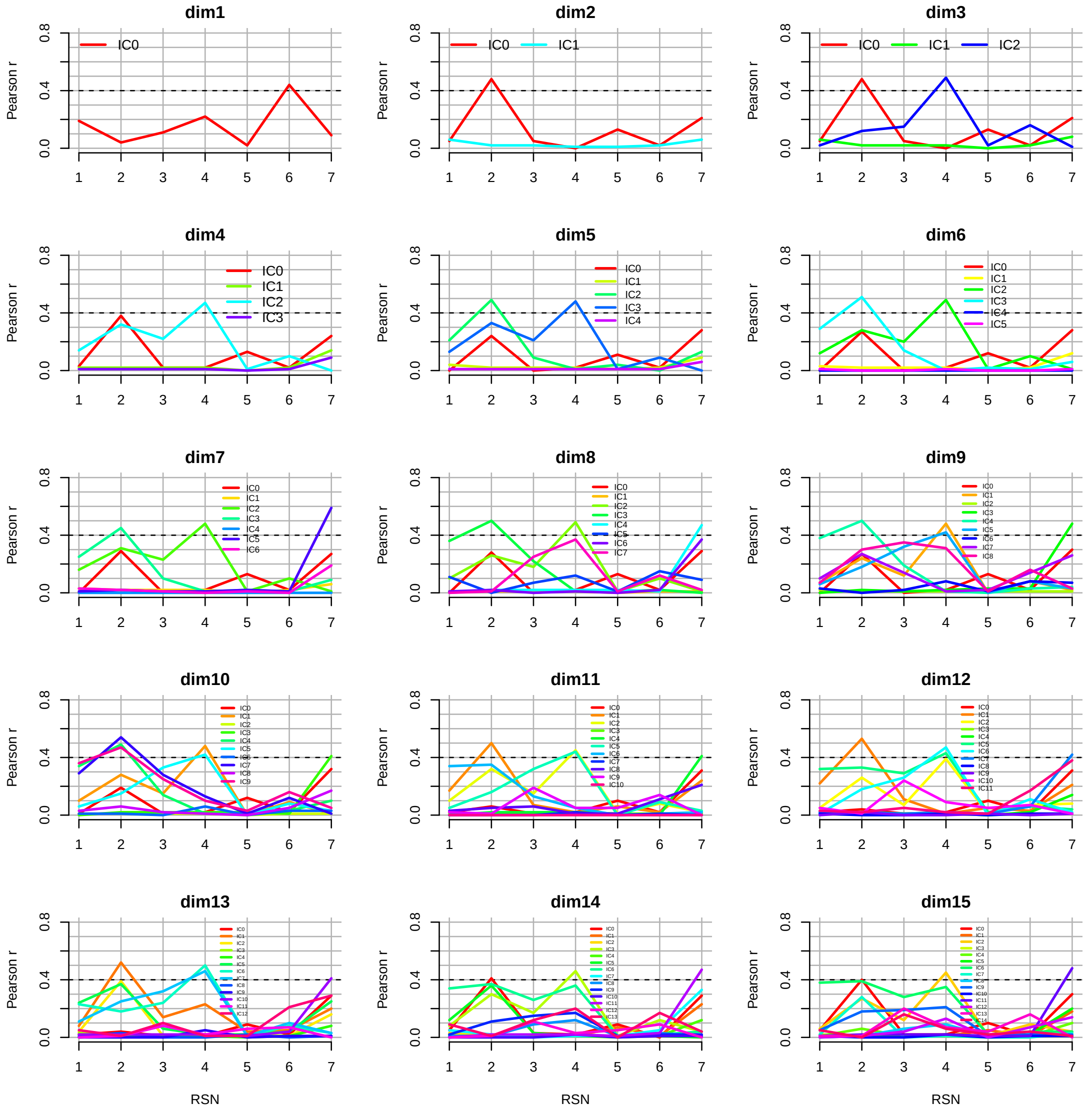

Figure S1: Overview of correlations between whole-brain FC maps and the 7 Yeo networks for each IC across all 15 dimensions tested. Note that three Yeo networks (2,4,7) appear frequently across all dimensions and that the lowest dimension at which these three networks are detected with good strength and separability is dimension 7.

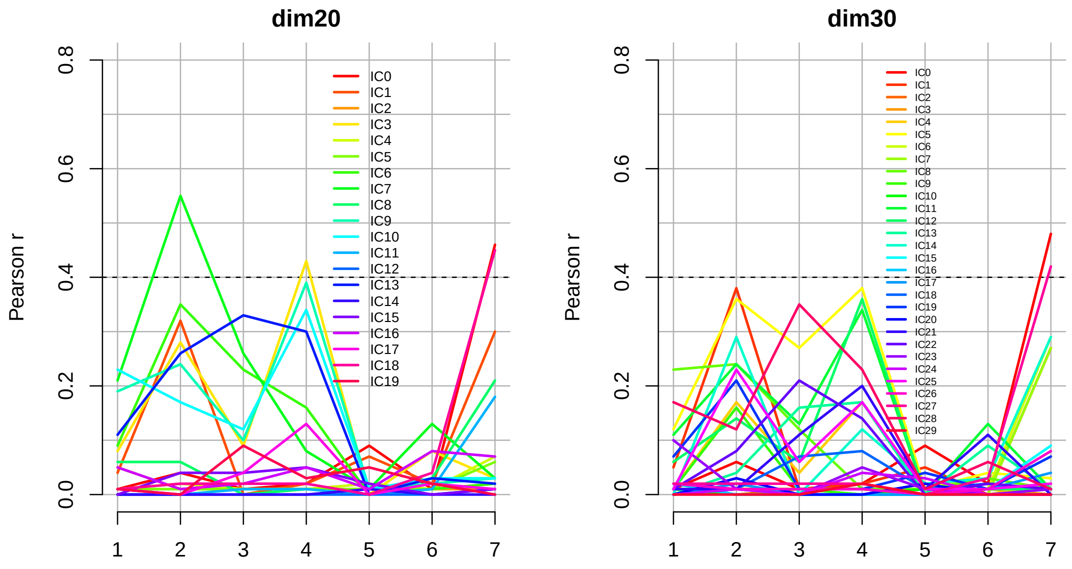

Figure S2: Correlations between independent components derived from srICA at dimensions 20 and 30 and the 7 different resting-state networks. Note how even at increased dimensions, networks 2 (somatomotor), 4 (ventral attention) and 7 (default mode) seem most frequently detected, albeit less reliably.

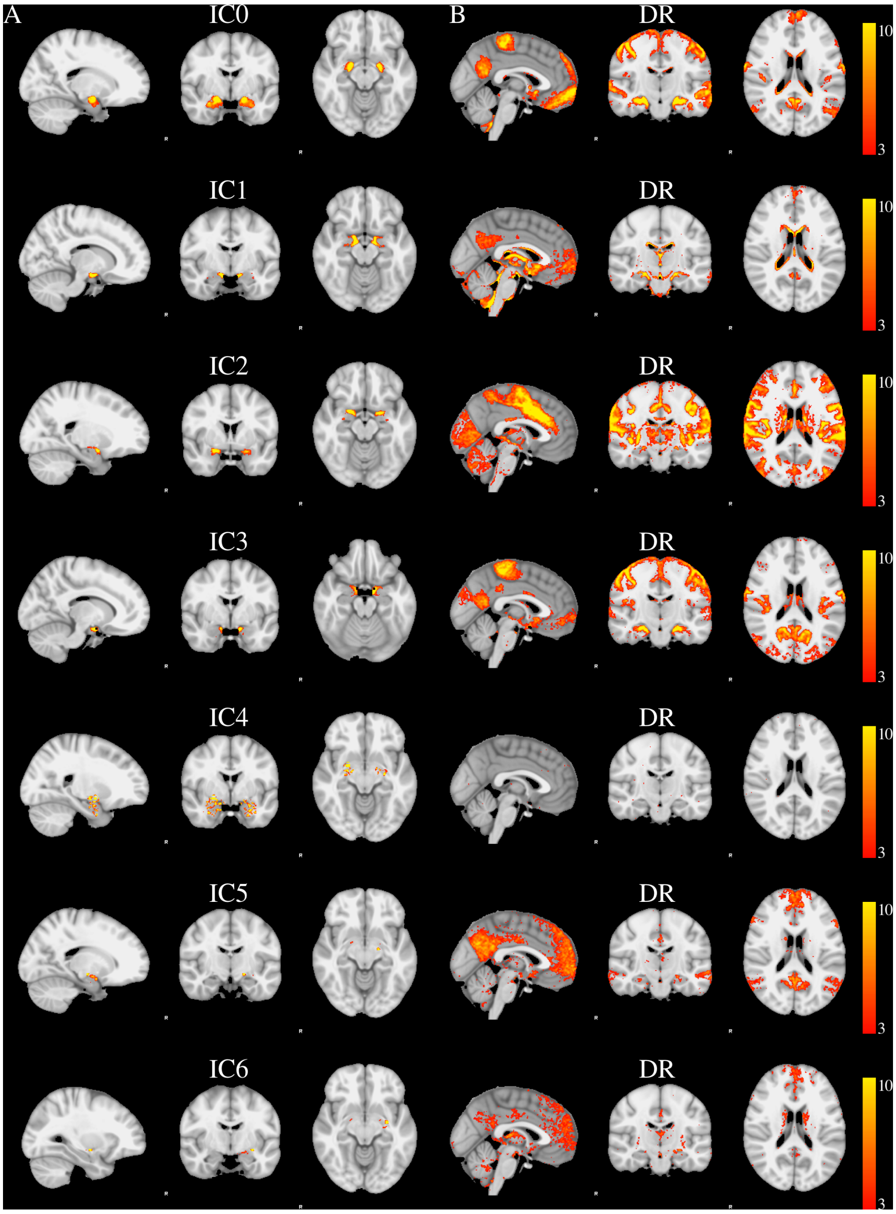

Figure S3: Overview of the spatial location of detected ICs inside the amygdala (left column, A), as well as their corresponding whole-brain FC maps (right column, B) for all ICs with dimension 7. Note ICs 2 (Ventral Attention), 3 (Somatomotor), and 5 (default mode) were detected by the algorithm as having a strong and unique relationship with the Yeo networks (see main manuscript text for details).

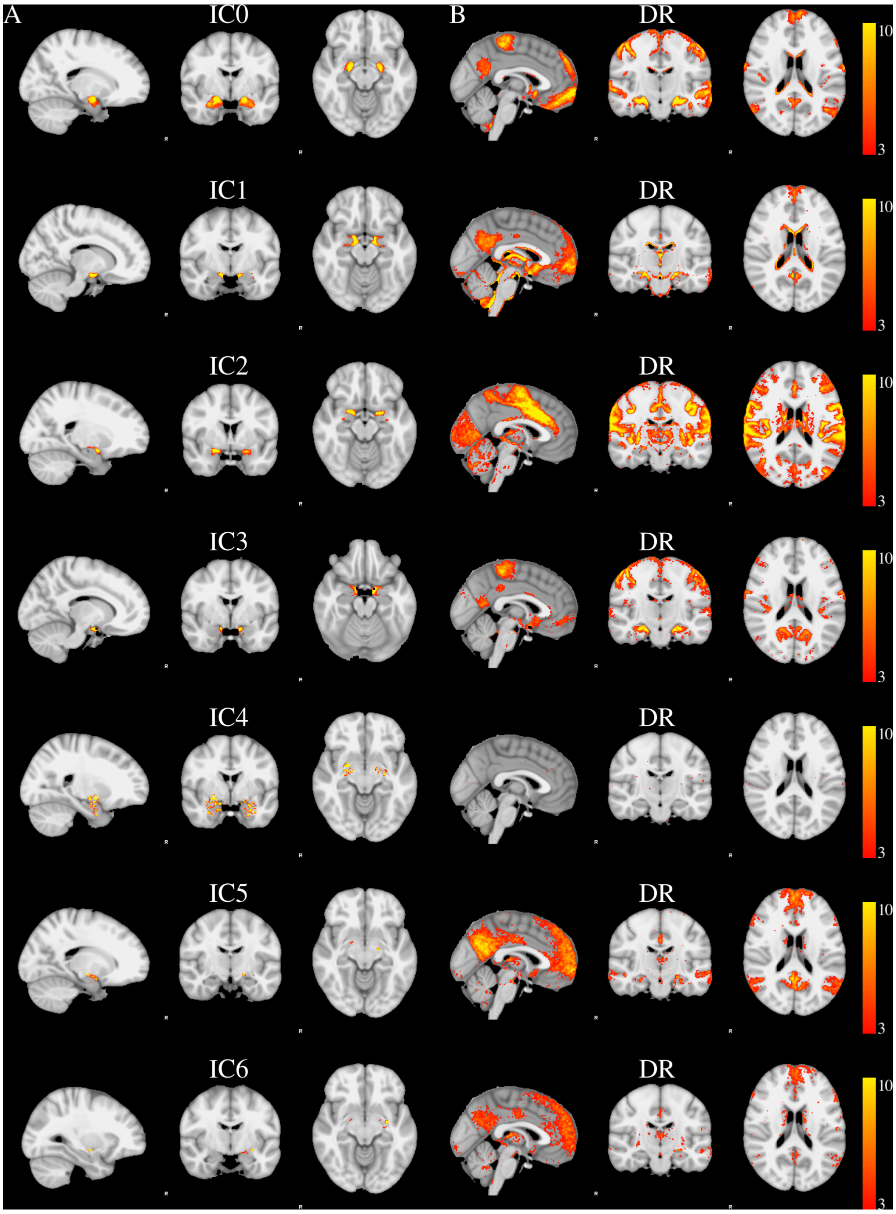

Figure S4: Overview of results in the validation dataset. Note the high similarity between the obtained functional connectivity for ICs 2, 3 and 5 in the validation and test datasets (cf., Supplementary Figure S3).
